# Self-Reflection as a Starting Point: Observations in Global Health Research

**DOI:** 10.9745/GHSP-D-23-00381

**Published:** 2024-12-20

**Authors:** Wouter Bakker, Thomas van den Akker, Jelle Stekelenburg

**Affiliations:** a Athena Institute, VU University, Amsterdam, The Netherlands.; b Department of Obstetrics and Gynecology, Leiden University Medical Centre, Leiden, The Netherlands.; c Department of Health Sciences, Global Health Unit, University Medical Centre Groningen/University of Groningen, Groningen, The Netherlands.; d Department of Obstetrics and Gynecology, Leeuwarden Medical Centre, Leeuwarden The Netherlands.

## Abstract

As researchers in global health, we reflect on the inequities in our work and our own struggles with these inequities and suggest some points to consider to address them in future global health work.

## Key Messages

- Researchers in global health research should continuously reflect on their roles and positions and have a responsibility to address the inequities observed in the research community.
- We discuss our observations concerning inequity in global health research and our struggles with these inequities.
- We suggest several ways to address some of the inequities, such as revising the author ranking structure and redistributing resources in global health research.

## INTRODUCTION

In 2020, Seye Abimbola pictured a world in which global health will be completely decolonized, with research led by those closest to the largest global problems and academic institutes in the Global South as influential as in the Global North.^1^ Unfortunately, such a world is still far away. As researchers and physicians with experience in global health research in collaborative settings, we reflect on our work and the current state of collaboration between the global North and South.

We were trained as Physicians Global Health and Tropical Medicine, a post-medical school training in the Netherlands—unique for any high-income setting—that, among other career perspectives, prepares doctors to work as generalists in low-resource settings.^2^ Trainees are encouraged to reflect on their role and position and engage in clinical evaluations and research, aiming to solve locally emerging questions. Consequently, many combine clinical work and scientific research, sometimes leading to a doctoral degree.^3^ This practice has led to substantial collaborative research networks, with 2 endowed chairs in the field of safe motherhood and sexual reproductive health and rights in the country.

Within this collaboration, there are ample opportunities for researchers from the Global South to lead or participate in research projects and obtain further education and academic degrees at a Dutch university or elsewhere. However, while such opportunities are increasing, there is still considerable inequity between researchers from Global North and South in terms of authorship, funding opportunities, possibilities to acquire academic degrees, and the perceived value of such degrees in the eyes of global academia if obtained from a university in the Global South as opposed to the Global North.

In this article, we discuss our observations and reflect on our own practice. Throughout this article, although we frequently use the terms North, South, local, and foreign, we do not want to create division by using this vocabulary. We realize that the term “local” might carry a negative connotation, which is not our intention. Our views are mere reflections of our own thoughts and discussions and are not aimed at any specific person or institution.

## OBSERVATIONS PERTAINING TO INEQUITY IN GLOBAL HEALTH RESEARCH

First, imbalances in authorship persist despite increased attention to performing demand-driven research in context, whereby local engagement is now a prerequisite for many projects and journals. A review on maternal health research in low- and middle-income countries showed an initial increase in articles led by authors from low- and middle-income countries, which has stagnated in more recent years. Considerable differences between continents and countries were identified, whereby for research from 10 countries, less than 25% of publications had a first author from those countries.^4^ An analysis by a senior editorial board member of *BMC Pregnancy and Childbirth* on their submitted articles from 7 sub-Saharan African settings (N=609) also showed substantial imbalances in authorship in collaborative projects. Apart from research from Ethiopia, complete Global South authorship without involvement from Global North collaborators was rare (0%–17.5%). It was also infrequent to find a senior/last author within a collaborative group from the Global South (4.8% for Rwanda to 72.4% for Ethiopia). First authors from the Global South ranged from 39.6% (Malawi) to 70.9% (Ethiopia) (personal communication, Jos van Roosmalen, May 2023). A systematic review on authorship for infectious disease research in Africa between 1980 and 2016 showed similar imbalances, with only 49.8% of first authors and 41.3% of last authors affiliated with African institutions.^5^ In 93.6% of the articles, at least 1 African author was involved, a promising increase compared to the past.

Imbalances in authorship persist despite increased attention to performing demand-driven research in context.

At times, we ourselves have also struggled with these authorship imbalances and have published studies from settings in the Global South with first and/or last authors from the Global North. Although these authors from the Global North had been working for substantial time frames in the Global South and in close collaboration with Global South authors, some of our work nonetheless has compounded the inequity we aim to address. In other words, although we consider it imperative to stimulate and contribute to Global South-led and -supervised projects, we ourselves have been part of the problem of Global North privilege.

Second, we see unfairness when it comes to opportunities for researchers, for example, in pursuing further education and academic degrees. As clinicians and researchers from a high-resource setting, we had ample opportunities to start and engage in projects in settings outside of The Netherlands. This has led to additional questions and self-reflection. Who are we to study and write about settings we may have only worked in for a few years? Why did we get the chance to pursue further education or defend a PhD dissertation based on this work while no longer being confronted daily with the problems described in the dissertation after returning to our home countries?

We love our work but are painfully aware that we function within institutions that, in the global academic rat race, have an incentive to keep the inequity alive. We are, of course, grateful for the opportunities we received and have embraced them. However, maybe we did not always use these opportunities to initiate meaningful change for the betterment of our Global South colleagues. Over time, we have tried to adapt and increase the support and opportunities for them. At the same time, sometimes, starting a research project only seems possible by going through a difficult web of applications, grants, and scholarships that are available only for the fortunate few, with disproportionate representation from Global North researchers and higher chances of success for them. A recent *Lancet* commentary described how an average grant application is estimated to take 38 full-time days to develop, sometimes as much time as it takes to perform the project itself.^6^

Third, we observe an imbalance between conducting research and disseminating results. Gathering information on clinical practice is useful for the study setting itself, and dissemination can inspire others and contribute to scientific advancement. While it can be very helpful to document findings in a publication, care should be taken to first disseminate and discuss the findings within the studied setting. The publication process, crucial to filter for quality, can be tedious and expensive and certainly more difficult to navigate for those in low-resource settings. Open-access publication is now becoming the standard way of publishing, which hopefully will increase access to scientific resources, but it is also costly for researchers, especially in collaborative projects, where fee waivers are not always applicable. We realize that we have also placed emphasis on the international publication of results. While international dissemination is crucial to enable learning across settings, we must not forget the initial context-specific questions and local needs from which research projects arise and ensure that these are addressed.

Fourth, researchers from low-resource settings often combine research with (sometimes inescapable) clinical activities and lack a supportive administrative or human resource management system. These researchers rarely have access to funding or institutional publishing agreements. While a combination of clinical work and (operational) research is very useful and encouraged, it requires substantial endurance and motivation of the clinical staff and is not prioritized in all settings. In contrast, we have witnessed clinical settings sometimes be drained of staff deployed to research projects, often led by academia from the Global North, putting additional pressure on the remaining staff to perform the clinical duties. Although we, in our projects, have aimed to keep the combination of research and clinical duties as optimal as possible for all involved and have attempted to refrain from having research interfere with clinical practice, we are aware that we might not always have succeeded in these aims.

## WAYS FORWARD

Our observations imply renewed opportunities to start conversations, as Abimbola encouraged us to do.^7^ Additionally, we suggest to the global health research community 4 ways forward for current and future projects.

First, we need to consider revisiting current ideas on “author ranking” to ensure equal opportunity for authors from the Global North and South. We should consider refraining from assigning a particular value to first and last authors in global health research. Adjusting the academic requirements for appointments or degrees, which is now often based on the number of first or last author publications, could assist global health researchers in achieving equality. We should strive for a more inclusive model in which other positions in the author byline are valued equally and an expression of collaboration in terms of authorship is key. More focus should be placed on the collaboration rather than on individual achievements.

Second, a larger proportion of the US$240 billion spent annually on health research should be allocated to locally led demand-driven research.^8^ There has not been much change since the identification of the 90/10 gap over 30 years ago, whereby less than 10% of global research investments were oriented toward 90% of the global health burden. Currently, only a fraction of the annually spent health research budget is directly relevant to health care in low-resource settings, and most of the expenditure is on internationally prioritized disease-specific research initiated and led by researchers from the Global North. Even if only a small fraction of the global health research investment would be shifted toward locally led demand-driven research in low-income countries, this could lead to fundamental changes in the research landscape. Bekele et al. suggest the grant structure be revised with earlier appraisal, detailed feedback, increased transparency, and the option to apply for subsequent calls.^6^ This could encourage researchers from anywhere to apply for funding.

Third, we should focus research first on benefiting the setting where it was performed and then to serve as inspiration for those in other settings. With this in mind, we should value participatory action research and transdisciplinary research, particularly research contributing to cross-border knowledge exchange and the formation of long-term international partnerships. Projects should always be adapted to the context in which they are implemented. To ensure this, as researchers we need to remain critical of our work, continuing to self-reflect on our personal roles and try to act on our reflections. This reflection should be routine within every research team and have an important place in research education. This also requires research teams to critically assess their collaboration and author roles. Reflecting on our own projects, we acknowledge that we also have made mistakes. We did not always act on local needs and questions. We joined in the struggle for author rankings and the academic importance attributed to these. At times, our research projects put an increased burden on health professionals and their clinical duties. Our learning has gradually led to extended collaboration in subsequent projects, adjustment of roles and responsibilities, advice and training of (research) students, and further adaptation of research to fit the context where it takes place.

We should focus research first on benefiting the setting where it was performed and then to serve as inspiration for those in other settings.

Fourth, stronger bonds between universities might create additional opportunities to acquire master’s or doctoral degrees in a larger number of locations, keeping researchers from having to uproot and travel all over the world and have more support or opportunities close by. Academic institutions in the Global South must be strengthened. This is key to the completion of “decolonizing” global health research and requires equal access to funds and resources and equal validation of institutions in different regions. Sustainable integration of clinical work and research in these institutions can also help prevent the draining of clinical staff who are supported by hospital management and research support staff and have equal access to scientific resources (e.g., literature and software). The balance between clinical activities and research should be closely monitored.

These points summarize key areas of focus, although the list of challenges and possible solutions is certainly longer. Therefore, we invite fellow researchers and health care professionals involved in global health to contribute and keep the conversation going. Constantly being aware of our positions and roles is a prerequisite for researchers. We need to work harder if we are to achieve the decolonized global health research world that Abimbola so hopefully envisioned for us.

## Notes

**Peer Reviewed**

**First Published Online:** November 20, 2024.
